# Supplementary figures and images for: Integrated RNA-seq and scRNA-seq to explore the biological mechanisms of mitophagy-related genes in ulcerative colitis
Source: PLoS One. 2026 Apr 20;21(4):e0346974. doi: 10.1371/journal.pone.0346974 (PMC13095012; doi:10.1371/journal.pone.0346974)

## Supplementary materials

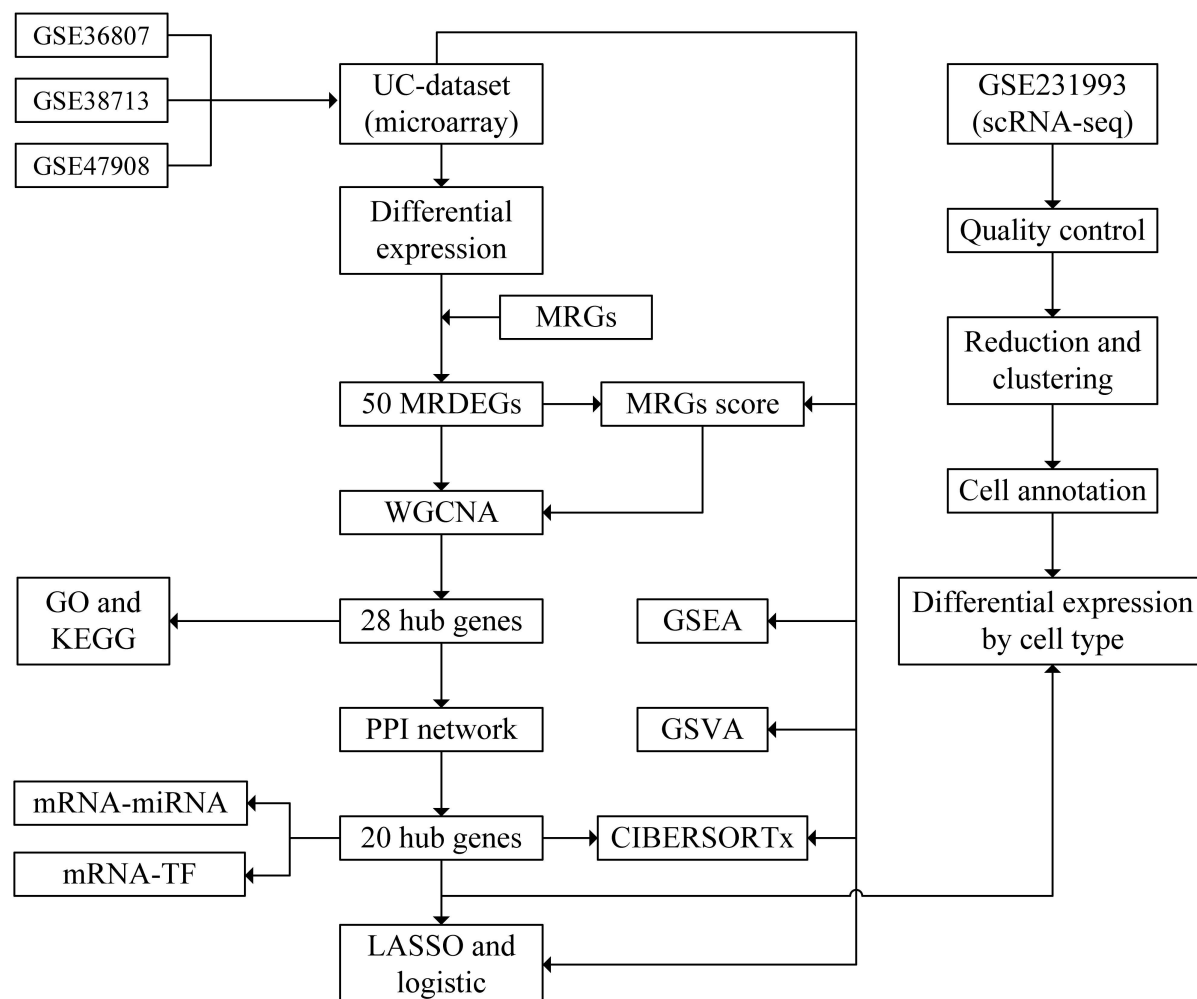

**Figure S1. Overall design and flowchart.**

Supplement: S1 Fig — (PDF) [file pone.0346974.s001.pdf]

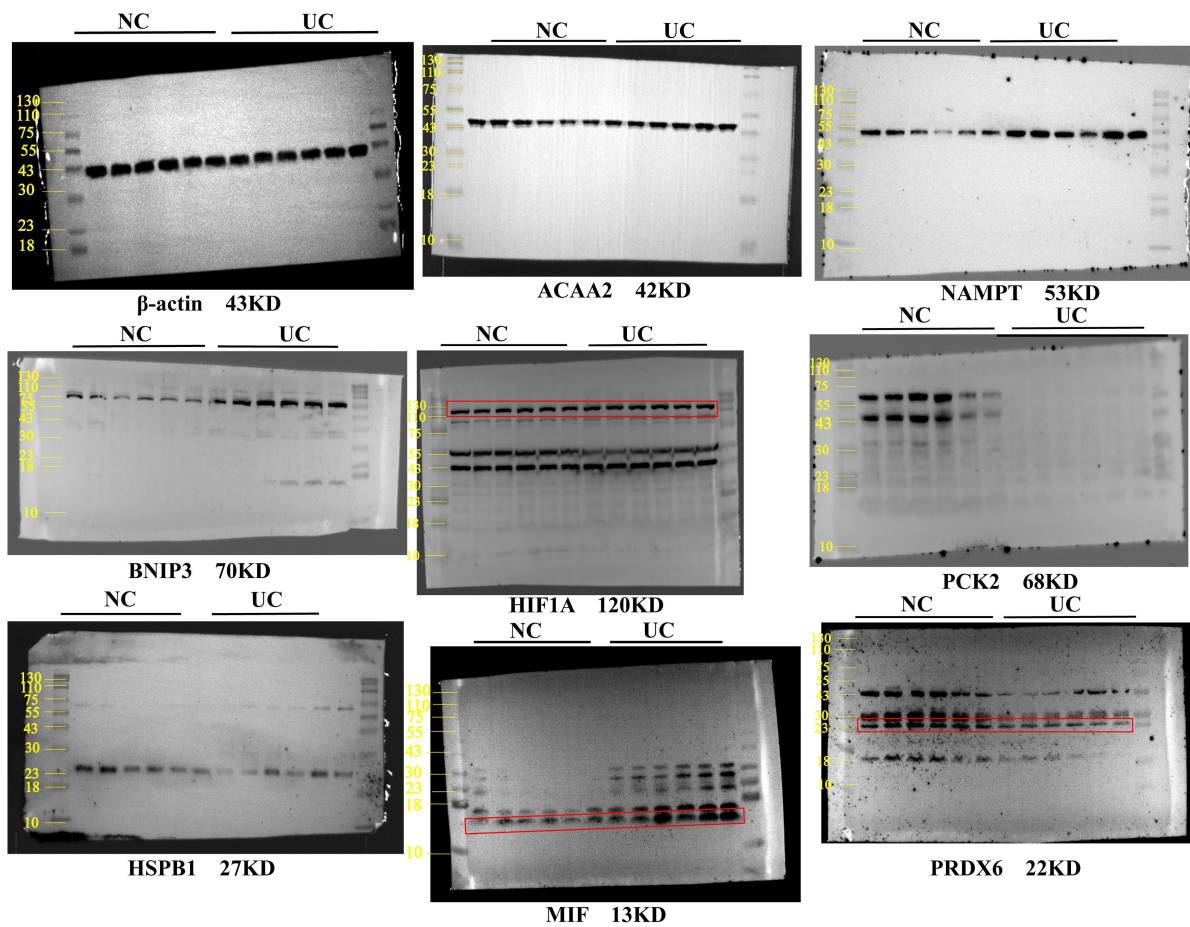

**Figure S4. Original protein images**

Supplement: S4 Fig — (PDF) [file pone.0346974.s004.pdf]

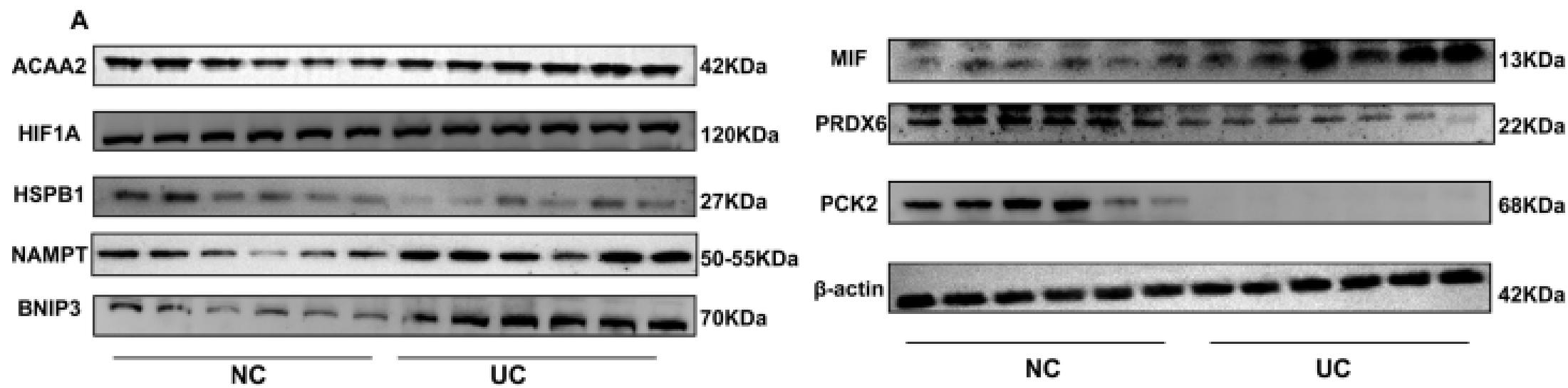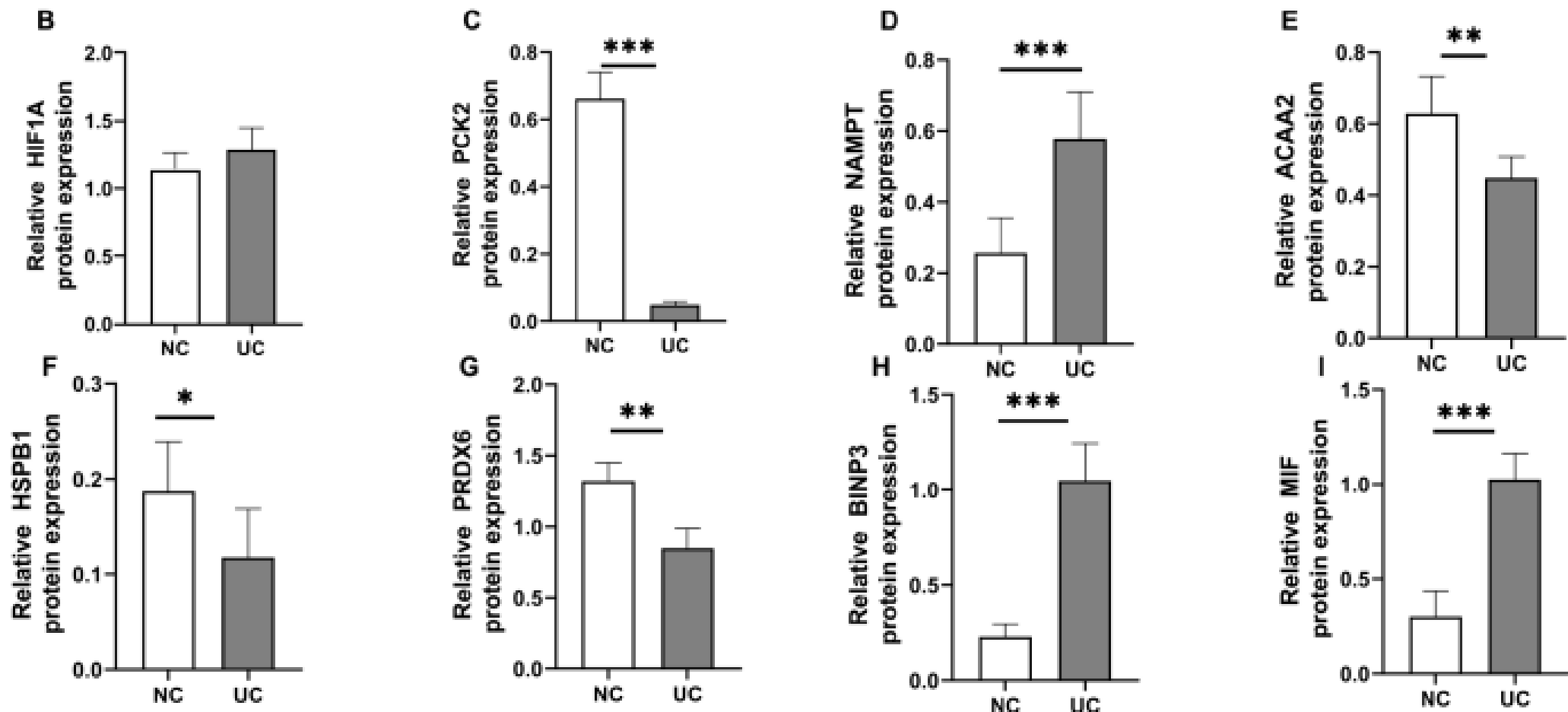

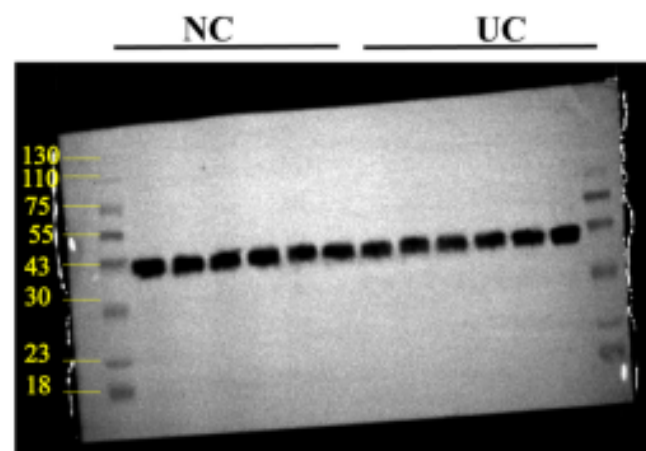

**β-actin 43KD**

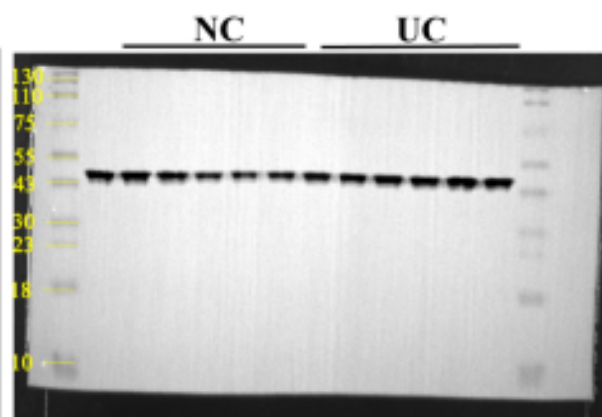

**ACAA2 42KD**

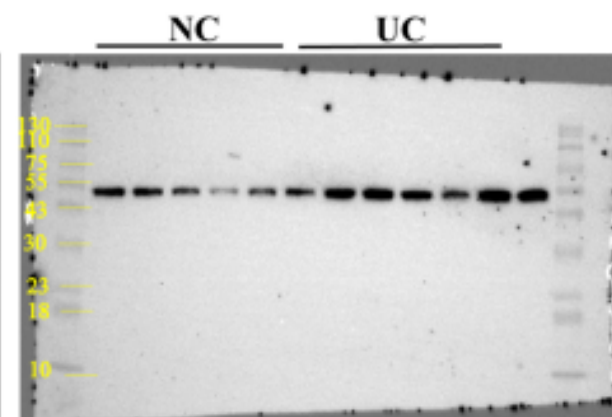

**NAMPT 53KD**

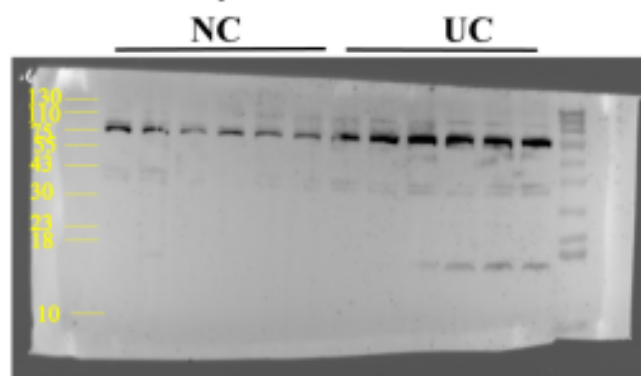

**BNIP3 70KD**

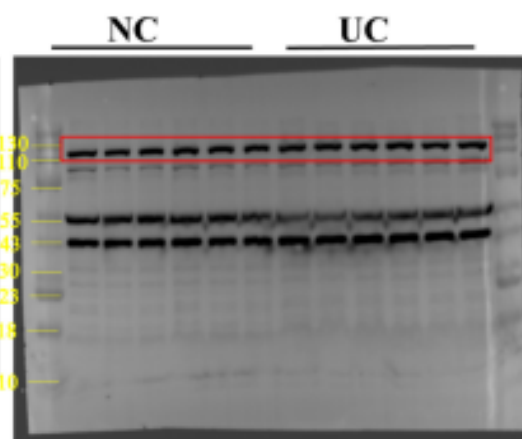

**HIF1A 120KD**

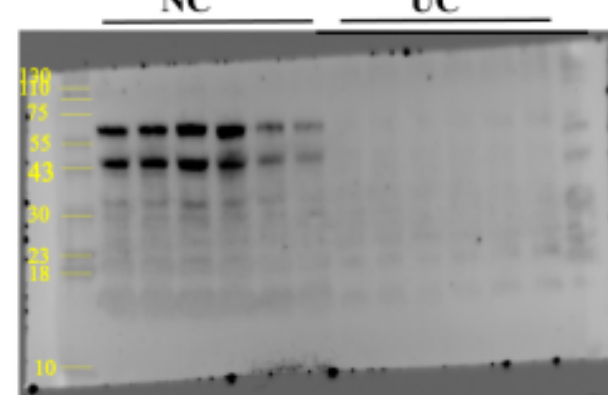

**PCK2 68KD**

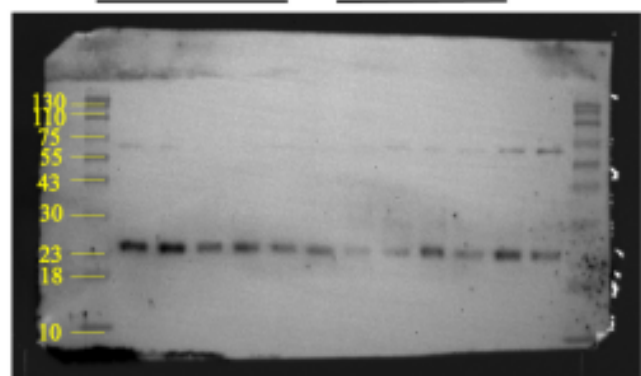

**HSPB1 27KD**

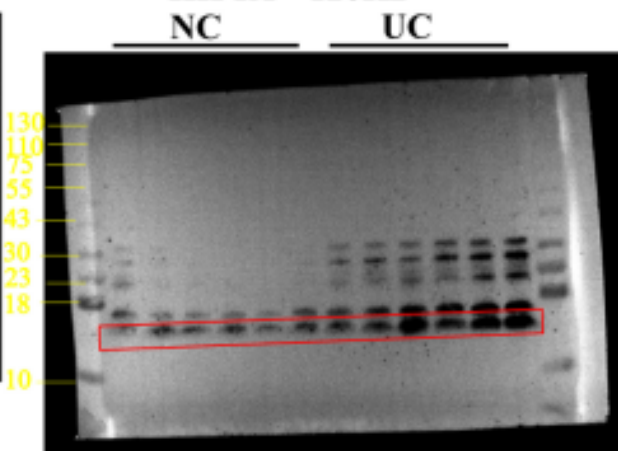

**MIF 13KD**

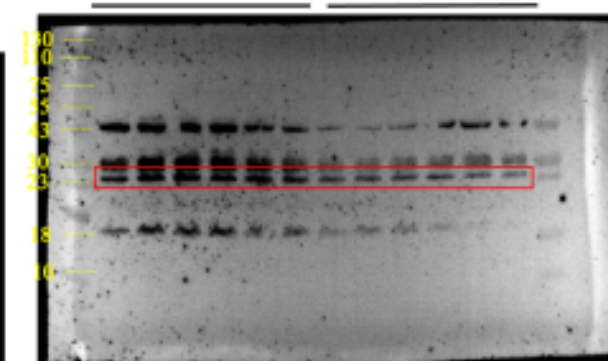

**PRDX6 22KD**

Supplement: S1 File — Original protein images and protein statistical chart. (PDF) [file pone.0346974.s012.pdf]
