# Supplementary material for: Integrated RNA-seq and scRNA-seq to explore the biological mechanisms of mitophagy-related genes in ulcerative colitis
Source: PLoS One. 2026 Apr 20;21(4):e0346974. doi: 10.1371/journal.pone.0346974 (PMC13095012; doi:10.1371/journal.pone.0346974)
Supplement: S2 Fig — (PDF) [file pone.0346974.s002.pdf]

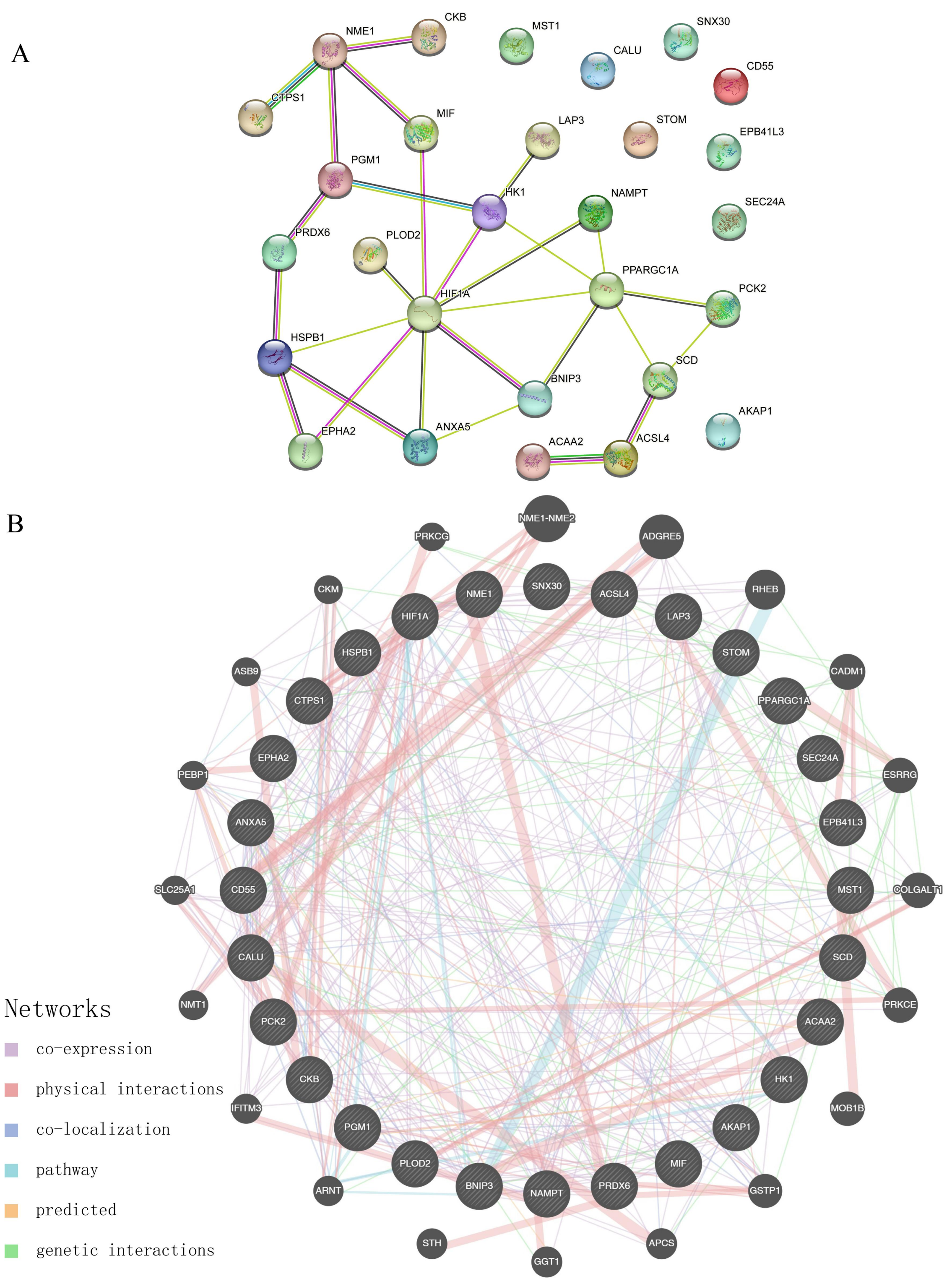

Figure S2. PPI interaction network. (**A**) Network of central genes regarding protein-protein interactions. (**B**) GeneMANIA website predicts interaction networks of genes with similar functions, acting as hub genes. PPI, protein-protein interaction.
