# Supplementary material for: Integrated RNA-seq and scRNA-seq to explore the biological mechanisms of mitophagy-related genes in ulcerative colitis
Source: PLoS One. 2026 Apr 20;21(4):e0346974. doi: 10.1371/journal.pone.0346974 (PMC13095012; doi:10.1371/journal.pone.0346974)
Supplement: S3 Fig — (PDF) [file pone.0346974.s003.pdf]

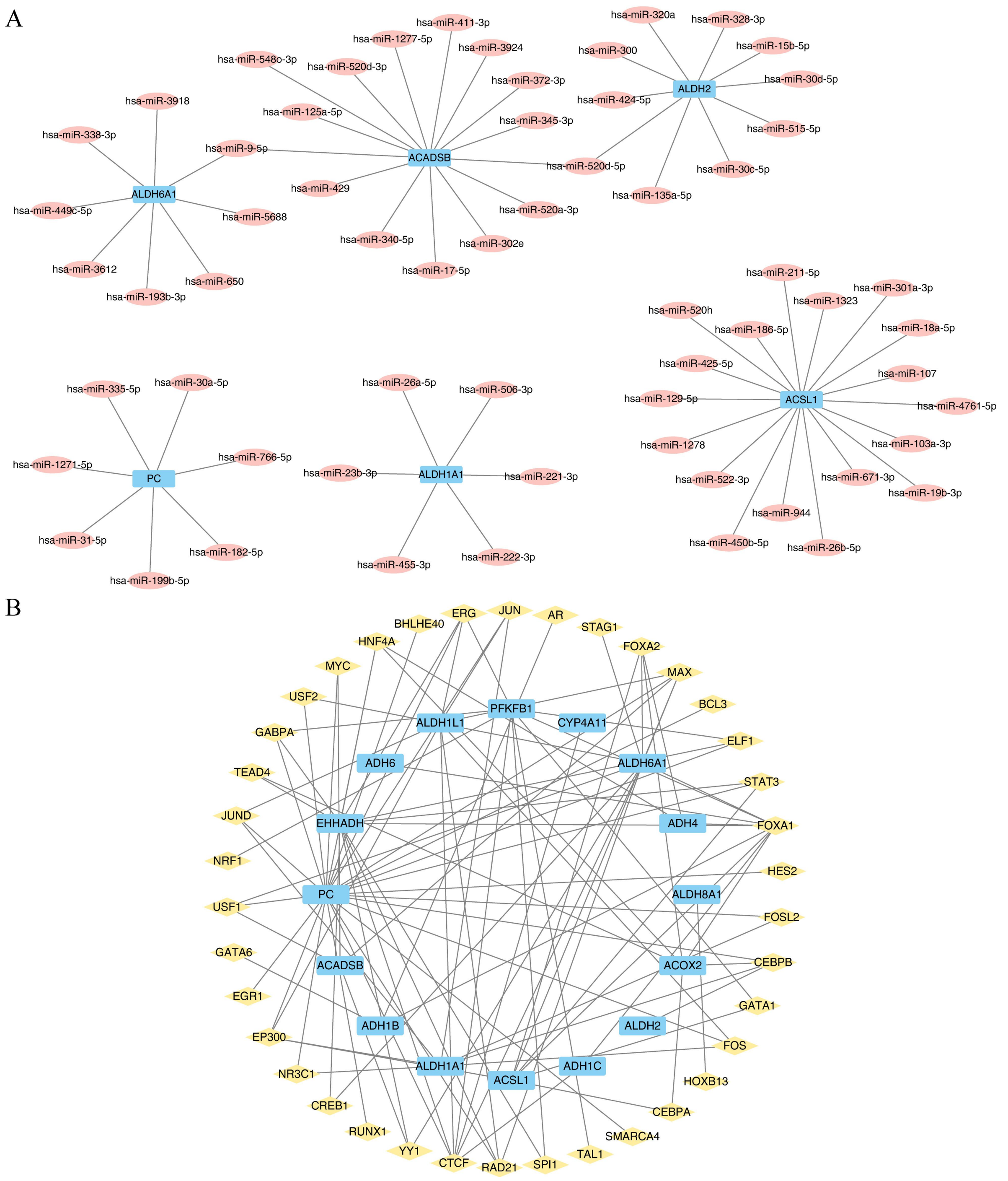

Figure S3. miRNA of hub genes, TF-prediction network. (**A**) mRNA-miRNA prediction network for hub genes. Blue rectangles in prediction network represent mRNAs, red ovals represent miRNAs, and interaction data are derived from ENCORI database. (**B**) mRNA-TF prediction network for hub genes. Blue rectangles in prediction network represent mRNA, yellow diamonds represent TF, and interaction data are derived from ChIPBase 3.0 database. TF, transcription factor.
