# Supplementary material for: Integrated RNA-seq and scRNA-seq to explore the biological mechanisms of mitophagy-related genes in ulcerative colitis
Source: PLoS One. 2026 Apr 20;21(4):e0346974. doi: 10.1371/journal.pone.0346974 (PMC13095012; doi:10.1371/journal.pone.0346974)
Supplement: S1 Table — (PDF) [file pone.0346974.s005.pdf]

Table S1. Information of datasets.

Items	GSE36807	GSE38713	GSE47908	GSE231993
Platform	GPL570	GPL570	GPL570	GPL18573
Squencing type	Expression profiling by array	Expression profiling by array	Expression profiling by array	Expression profiling by high throughput sequencing
Species	Homo sapiens	Homo sapiens	Homo sapiens	Homo sapiens
Disease	UC	UC	UC	UC
Tissue	colon	colon	colonic mucosal	colon region
Samples in disease group	15	30	45	4
Samples in control group	7	13	15	4
Reference	[1]	[2]	[3]	/
