# Supplementary material for: Integrated RNA-seq and scRNA-seq to explore the biological mechanisms of mitophagy-related genes in ulcerative colitis
Source: PLoS One. 2026 Apr 20;21(4):e0346974. doi: 10.1371/journal.pone.0346974 (PMC13095012; doi:10.1371/journal.pone.0346974)
Supplement: S2 Table — (PDF) [file pone.0346974.s006.pdf]

Table S2. List of gene symbol of MRGs.

	Gene symbol		Gene symbol		Gene symbol
1	AAAS	263	HMGB1	525	RNF41
2	AARS2	264	HNRNPA2B1	526	ROCK2
3	ABAT	265	HNRNPA3	527	RPL11
4	ABCB10	266	HNRNPD	528	RPL12
5	ABCD3	267	HNRNPDL	529	RPL13A
6	ABCE1	268	HNRNPH1	530	RPL15
7	ACAA2	269	HNRNPK	531	RPL18
8	ACBD3	270	HNRNPM	532	RPL18A
9	ACIN1	271	HNRNPU	533	RPL21
10	ACSF3	272	HPF1	534	RPL28
11	ACSL4	273	HRAS	535	RPL35A
12	ACTB	274	HS1BP3	536	RPL38
13	ACTBL2	275	HS2ST1	537	RPL4
14	ACTL6A	276	HSP90AA1	538	RPL7
15	ACTR10	277	HSP90AB1	539	RPL8
16	ACTR1A	278	HSP90B1	540	RPL9
17	ACTRT1	279	HSPA1A	541	RPLP0
18	ADH5	280	HSPA1L	542	RPS15A
19	ADSS2	281	HSPA4	543	RPS16
20	AIFM1	282	HSPA5	544	RPS18
21	AKAP1	283	HSPA8	545	RPS2
22	AKAP11	284	HSPA9	546	RPS26
23	AKR1E2	285	HSPB1	547	RPS27A
24	AKT1	286	HSPD1	548	RPS27L
25	ALB	287	HTRA2	549	RPS3
26	ALDH18A1	288	HTT	550	RPS3A
27	ALDH2	289	HUWE1	551	RPS5
28	ALDH3A2	290	IDH2	552	RPS9
29	ALDH7A1	291	IDH3A	553	RPTOR
30	AMBRA1	292	IDH3B	554	RRAGA
31	AMFR	293	IDH3G	555	RRAGC
32	ANKRD13A	294	IGF1	556	RTN3
33	ANO6	295	IGF2R	557	RTN4
34	ANXA2	296	ILF2	558	RUVBL1
35	ANXA5	297	ILK	559	SAE1
36	APAF1	298	IMMT	560	SAMM50
37	APBB1	299	IRF3	561	SAR1A
38	APP	300	IRGM	562	SAR1B
39	ARFIP2	301	ITCH	563	SARS1
40	ARIH1	302	ITPR2	564	SCAMP3
41	ARIH2	303	JUP	565	SCD
42	ARL6IP5	304	KDELR1	566	SCO2
43	ARL8B	305	KEAP1	567	SCRN1
44	ARMCX2	306	KHNYN	568	SDHA
45	ARMCX3	307	KIAA0232	569	SEC16A
46	ARPC4	308	KIF1A	570	SEC22B
47	ATAD3A	309	KPNA5	571	SEC23A

48	ATAD3B	310	KRAS	572	SEC24A
49	ATG12	311	KRT15	573	SEC24B
50	ATG13	312	L2HGDH	574	SEC61B
51	ATG14	313	LAP3	575	SEC62
52	ATG16L1	314	LBR	576	SEC63
53	ATG2A	315	LDHA	577	SEH1L
54	ATG2B	316	LMAN1	578	SENP3
55	ATG4A	317	LMCD1	579	SERPINB6
56	ATG4B	318	LMNA	580	SESN2
57	ATG4C	319	LMO7	581	SF3A1
58	ATG4D	320	LONP1	582	SF3A2
59	ATG5	321	LPCAT3	583	SGPL1
60	ATG7	322	LRBA	584	SH3GLB1
61	ATG9A	323	LRCH4	585	SHC1
62	ATM	324	LRPPRC	586	SHMT2
63	ATP1B1	325	LRRC59	587	SHPK
64	ATP5F1A	326	LRRK2	588	SIAH1
65	ATP5F1B	327	LYAR	589	SIAH3
66	ATP5IF1	328	MACROH2A1	590	SIRT1
67	ATP6AP1	329	MADD	591	SIRT2
68	ATP6V1A	330	MAGOHB	592	SIRT3
69	ATP6V1G1	331	MAP1A	593	SLC12A2
70	ATP7B	332	MAP1LC3A	594	SLC12A4
71	AUP1	333	MAP1LC3B	595	SLC12A6
72	B3GAT3	334	MAP1LC3B2	596	SLC25A24
73	BAG5	335	MAP1LC3C	597	SLC25A3
74	BAG6	336	MAP2K1	598	SLC25A4
75	BAK1	337	MAP2K2	599	SLC25A5
76	BAX	338	MAP2K3	600	SLC33A1
77	BCAS3	339	MAP2K7	601	SLC3A2
78	BCAT2	340	MAP3K7	602	SMAD2
79	BCKDK	341	MAPK1	603	SMARCAD1
80	BCL2	342	MAPK14	604	SMURF1
81	BCL2L1	343	MAPK15	605	SNAP47
82	BCL2L13	344	MAPK3	606	SNCA
83	BCS1L	345	MAPK8	607	SNX1
84	BECN1	346	MAPK9	608	SNX17
85	BET1	347	MARCHF5	609	SNX2
86	BIRC2	348	MATR3	610	SNX3
87	BLMH	349	MAVS	611	SNX30
88	BLOC1S1	350	MBOAT7	612	SNX4
89	BLTP3A	351	MCL1	613	SOAT1
90	BLVRA	352	MCM7	614	SPATA18
91	BNIP3	353	MDH1	615	SPATA33
92	BNIP3L	354	MEGF8	616	SPCS2
93	C1QBP	355	MFF	617	SPG21
94	C9orf72	356	MFN1	618	SPTAN1
95	CALCOCO2	357	MFN2	619	SPTBN1
96	CALU	358	MIEF1	620	SPTBN2
97	CAND1	359	MIF	621	SQSTM1

98	CANX	360	MIOS	622	SRC
99	CAPN1	361	MON1A	623	SREBF1
100	CAT	362	MON1B	624	SREBF2
101	CAV1	363	MON2	625	SRP54
102	CCDC47	364	MRI1	626	SRPRA
103	CCT2	365	MRPL37	627	SRPRB
104	CCT3	366	MRPL4	628	SRSF4
105	CCT4	367	MRPL44	629	ST7
106	CCT7	368	MRPS16	630	STARD7
107	CCZ1B	369	MRPS2	631	STEAP3
108	CD55	370	MRPS30	632	STIM1
109	CDC34	371	MRPS34	633	STK38
110	CDC37	372	MRPS7	634	STK4
111	CDK11A	373	MST1	635	STOM
112	CDK5RAP3	374	MT-ND6	636	STOML2
113	CDKAL1	375	MTARC1	637	STT3B
114	CDKN2A	376	MTARC2	638	STX10
115	CERS1	377	MTCH2	639	STX17
116	CFAP20	378	MTERF3	640	SUCLA2
117	CFL1	379	MTOR	641	SUN1
118	CHAF1B	380	MTPAP	642	SYNE2
119	CHCHD3	381	MTX1	643	TAB1
120	CHMP2A	382	MTX2	644	TAFAZZIN
121	CHMP5	383	MUL1	645	TALDO1
122	CHUK	384	MYH11	646	TARDBP
123	CISD1	385	MYH9	647	TAX1BP1
124	CISD2	386	MYLK	648	TBC1D15
125	CKAP4	387	MYO6	649	TBC1D17
126	CKB	388	NAA10	650	TBC1D22A
127	CLEC16A	389	NAA16	651	TBC1D5
128	CLINT1	390	NADSYN1	652	TBK1
129	CLPB	391	NAMPT	653	TCHP
130	CLUH	392	NAXE	654	TCP1
131	CNOT4	393	NBR1	655	TDRKH
132	CNP	394	NCAPD2	656	TEX10
133	COASY	395	NDC1	657	TFE3
134	COG6	396	NDUFA10	658	TFEB
135	CORO1A	397	NDUFA9	659	TFRC
136	CORO1C	398	NDUFAF4	660	TGFB1
137	CPT1A	399	NDUFS1	661	THOP1
138	CREB1	400	NDUFS7	662	TIGAR
139	CRNKL1	401	NDUFV1	663	TIMM23
140	CSE1L	402	NEPRO	664	TIMM44
141	CSNK2A1	403	NFE2L2	665	TMEM11
142	CSNK2A2	404	NFKB1	666	TMEM43
143	CSNK2B	405	NIBAN2	667	TMPO
144	CTNND1	406	NIPSNAP1	668	TMX3
145	CTPS1	407	NIPSNAP2	669	TNFAIP8L1
146	CWC22	408	NKAP	670	TOM1
147	CYB5R1	409	NLRP3	671	TOMM20

148	CYP20A1	410	NME1	672	TOMM22
149	CYP51A1	411	NME4	673	TOMM40
150	DAP3	412	NME6	674	TOMM5
151	DARS2	413	NPEPPS	675	TOMM6
152	DCD	414	NPLOC4	676	TOMM7
153	DCTN4	415	NR4A1	677	TOMM70
154	DCXR	416	NRF1	678	TOR1AIP1
155	DDX17	417	NUP155	679	TOR1AIP2
156	DDX39B	418	NUP205	680	TP53
157	DDX5	419	NUP93	681	TRAF2
158	DDX54	420	OAT	682	TRAF6
159	DEF8	421	OCIAD1	683	TRAP1
160	DERA	422	OGT	684	TRAPPC12
161	DGKE	423	OPA1	685	TRIM25
162	DHFR	424	OPTN	686	TRIM27
163	DHX29	425	OSBPL11	687	TRIM5
164	DHX38	426	OSBPL5	688	TRIP11
165	DHX57	427	OSBPL8	689	TSC2
166	DIAPH2	428	OSBPL9	690	TSG101
167	DISC1	429	PAICS	691	TSPO
168	DNAJC3	430	PAK2	692	TUBA1C
169	DNM1L	431	PARK7	693	TUBA8
170	DPM1	432	PARL	694	TUBB
171	DPYSL5	433	PARP1	695	TUBB2B
172	DSG2	434	PCK2	696	TUBB4A
173	DSP	435	PCNA	697	TUBB4B
174	EARS2	436	PDHA1	698	TUBB6
175	EDC3	437	PDIA6	699	TUBG1
176	EDC4	438	PDK1	700	TUFM
177	EEF1A1	439	PDK2	701	TXN
178	EEF1G	440	PELO	702	U2AF1
179	EEF2	441	PEX13	703	U2AF2
180	EIF2AK2	442	PFAS	704	UBA1
181	EIF2B1	443	PFKP	705	UBA52
182	EIF2S1	444	PGAM1	706	UBAP2L
183	EIF3C	445	PGAM5	707	UBB
184	EIF3F	446	PGK1	708	UBC
185	EIF4A1	447	PGK2	709	UBE2D3
186	EIF4A2	448	PGM1	710	UBE2G2
187	EIF4A3	449	PHAF1	711	UBE2L3
188	EMC1	450	PHB1	712	UBE2N
189	EMC4	451	PHB2	713	UBE2Z
190	EMD	452	PHGDH	714	UBXN1
191	ENO1	453	PI4K2A	715	UBXN6
192	EPB41L3	454	PI4KB	716	UCHL1
193	EPHA2	455	PIGK	717	UGDH
194	EPRS1	456	PIGS	718	ULK1
195	EPS15	457	PIGT	719	UMPS
196	ERLIN2	458	PIK3C3	720	UQCRC1
197	ESYT1	459	PIK3R4	721	UQCRC2

198	ESYT2	460	PINK1	722	USP14
199	EWSR1	461	PIP4K2B	723	USP15
200	EXD2	462	PIP4K2C	724	USP30
201	FADS2	463	PITRM1	725	USP33
202	FADS3	464	PKM	726	USP35
203	FAF1	465	PLEC	727	USP36
204	FAF2	466	PLEKHA1	728	USP8
205	FAM20B	467	PLOD2	729	UTP18
206	FANCC	468	PLSCR1	730	UVRAG
207	FASN	469	PMPCA	731	VAC14
208	FASTKD5	470	PMPCB	732	VAMP7
209	FBL	471	PNO1	733	VAPA
210	FBXO7	472	POLR2H	734	VAPB
211	FBXW11	473	POLR3A	735	VCAM1
212	FBXW7	474	POR	736	VCP
213	FDPS	475	PPARGC1A	737	VDAC1
214	FEN1	476	PPFIBP1	738	VDAC2
215	FIG4	477	PPIB	739	VDAC3
216	FIS1	478	PPM1G	740	VIM
217	FKBP15	479	PPP3CA	741	VIPAS39
218	FKBP8	480	PRDX6	742	VPS13C
219	FLOT2	481	PREB	743	VPS13D
220	FNIP2	482	PRKAA1	744	VPS16
221	FOXO3	483	PRKAA2	745	VPS26A
222	FTMT	484	PRKACA	746	VPS26B
223	FUNDC1	485	PRKCD	747	VPS26C
224	FUS	486	PRKCI	748	VPS29
225	FXR2	487	PRKD2	749	VPS35
226	FYCO1	488	PRKD3	750	VPS37A
227	GABARAP	489	PRKDC	751	VPS41
228	GABARAPL1	490	PRKN	752	VPS45
229	GABARAPL2	491	PRPF8	753	WARS1
230	GANAB	492	PSMB2	754	WASHC4
231	GAPDH	493	PSMD8	755	WDR26
232	GBA1	494	PSMG3	756	WDR41
233	GBF1	495	PTEN	757	WDR46
234	GCC1	496	PTPMT1	758	WDR6
235	GDAP1	497	PTPN1	759	WIPI1
236	GDI1	498	PTRH2	760	WIPI2
237	GDI2	499	PUM2	761	WWOX
238	GFAP	500	PYCR1	762	XIAP
239	GFPT2	501	RAB10	763	XPO1
240	GLS	502	RAB1B	764	YKT6
241	GLT8D1	503	RAB7A	765	YME1L1
242	GLUD1	504	RABGAP1	766	YWHAE
243	GLUL	505	RAD23B	767	YWHAH
244	GMPPA	506	RALGAPA1	768	YWHAZ
245	GOLGA4	507	RAN	769	ZC3HAV1
246	GOLGA5	508	RAP1B	770	ZDHHC13
247	GOLGB1	509	RB1CC1	771	ZFYVE1

248	GOPC	510	RCAN1	772	ZFYVE16
249	GORASP2	511	RCN1		
250	GOT2	512	RDH13		
251	GPD2	513	REEP5		
252	GPHN	514	RETREG1		
253	GRN	515	RFC3		
254	GSK3A	516	RFWD3		
255	HCCS	517	RHOT1		
256	HDAC3	518	RHOT2		
257	HDAC6	519	RIMOC1		
258	HDHD5	520	RIPK1		
259	HIF1A	521	RMC1		
260	HK1	522	RMDN3		
261	HK2	523	RNF121		
262	HLA-C	524	RNF31		

MRGs, mitophagy related genes.
