# Supplementary material for: Integrated RNA-seq and scRNA-seq to explore the biological mechanisms of mitophagy-related genes in ulcerative colitis
Source: PLoS One. 2026 Apr 20;21(4):e0346974. doi: 10.1371/journal.pone.0346974 (PMC13095012; doi:10.1371/journal.pone.0346974)
Supplement: S3 Table — (PDF) [file pone.0346974.s007.pdf]

Table S3. The primer information.

Gene	Sequence
SCD	FORWARD: CAGCCTGTTCGTTAGCACCTTC<br>REVERSE: GGGATTGAATGTTCTTGTCGTAGGG
PRDX6	FORWARD: CACGGGCAGGAACTTTGATGAG<br>REVERSE: CTTCGGAGAGGGTGGGAACTAC
PPARGC1A	FORWARD: TTCGCTGCTCTTGAGAATGGATATAC<br>REVERSE: TCGTCTGAGTTGGTATCTAGGTCTG
PCK2	FORWARD: TACCTCTGCTGCCACCAATCC<br>REVERSE: TTCCCAGTACACACCGCCATC
NME1	FORWARD: GGCGAGATCATCAAGCGGTTC<br>REVERSE: GCGGTCCTTCAGGTCAGTGTAG
NAMPT	FORWARD: TCTGGAAATCCTCTTGACACTGTATTG<br>REVERSE: CACGCCATCTCCTTGAATGACTC
MIF	FORWARD: CCAGAACCGCAACTACAGTAAGC<br>REVERSE: TTGGCAGCGTTCATGTCGTAATAG
HSPB1	FORWARD: AAGGAAGGCGTGGTGGAGATC<br>REVERSE: ACCTGGAGGGAGCGTGTATTTC
HK1	FORWARD: TGGACGGGACGCTCTACAAAC<br>REVERSE: GACAGGAGGAAGGACACGGTAC
HIF1A	FORWARD: CCACAACTGCCACCACTGATG<br>REVERSE: GCCACTGTATGCTGATGCCTTAG
CKB	FORWARD: CATTCAGACTGGCGTAGACAATCC<br>REVERSE: TGCTCATCACTGGGCTGGTAG
Bnip3	FORWARD: TGAGAAACACAAGCGTTATGAAGAAAG<br>REVERSE: CAGATGAGACAGTAACAGAGATGGAAG
ACAA2	FORWARD: ATAAGCAGAGAAGACTGTGACAGATAC<br>REVERSE: CATTGAAGTAGCCAGCCTCGTTAG
GAPDH	FORWARD:GCATGGCCTTCCGTGTTC<br>REVERSE:GATGTCATCATACTTGGCAGGTTT
