# Supplementary material for: Integrated RNA-seq and scRNA-seq to explore the biological mechanisms of mitophagy-related genes in ulcerative colitis
Source: PLoS One. 2026 Apr 20;21(4):e0346974. doi: 10.1371/journal.pone.0346974 (PMC13095012; doi:10.1371/journal.pone.0346974)
Supplement: S4 Table — (PDF) [file pone.0346974.s008.pdf]

Table S4. List of gene symbol of MRDEGs.

Symbol				
ABAT	ABCD3	ACAA2	ACSL4	AKAP1
ALDH18A1	ANXA5	ARMCX2	ARMCX3	BCAT2
BNIP3	CALU	CAV1	CD55	CKB
CORO1A	CPT1A	CTPS1	DERA	DSG2
DSP	EPB41L3	EPHA2	HIF1A	HK1
HSPB1	IDH2	LAP3	LPCAT3	LRRK2
MIF	MST1	NAMPT	NME1	PCK2
PDK2	PGM1	PLOD2	PPARGC1A	PRDX6
PREB	RCN1	SCD	SEC24A	SNX30
STOM	TUBB6	UGDH	USP30	VCAM1

MRDEGs, mitophagy related differentially expression genes.
