# Supplementary material for: Integrated RNA-seq and scRNA-seq to explore the biological mechanisms of mitophagy-related genes in ulcerative colitis
Source: PLoS One. 2026 Apr 20;21(4):e0346974. doi: 10.1371/journal.pone.0346974 (PMC13095012; doi:10.1371/journal.pone.0346974)
Supplement: S5 Table — (PDF) [file pone.0346974.s009.pdf]

Table S5. mRNA-miRNA interactions.

mRNA	miRNA	mRNA	miRNA	mRNA	miRNA
ACSL4	hsa-miR-16-5p	EPHA2	hsa-miR-5590-3p	PLOD2	hsa-miR-429
ACSL4	hsa-miR-19a-3p	HIF1A	hsa-miR-10b-5p	PLOD2	hsa-miR-3163
ACSL4	hsa-miR-181a-5p	HIF1A	hsa-miR-330-3p	PLOD2	hsa-miR-1277-5p
ACSL4	hsa-miR-181c-5p	HIF1A	hsa-miR-20b-5p	PPARGC1A	hsa-miR-200b-3p
ACSL4	hsa-miR-211-5p	HIF1A	hsa-miR-493-5p	PPARGC1A	hsa-miR-15b-5p
ACSL4	hsa-miR-216a-5p	HIF1A	hsa-miR-519d-3p	PPARGC1A	hsa-miR-30b-5p
ACSL4	hsa-miR-217	HIF1A	hsa-miR-580-3p	PPARGC1A	hsa-miR-146a-5p
ACSL4	hsa-miR-200b-3p	HIF1A	hsa-miR-584-5p	PPARGC1A	hsa-miR-299-3p
ACSL4	hsa-miR-130a-3p	HIF1A	hsa-miR-656-3p	PPARGC1A	hsa-miR-146b-5p
ACSL4	hsa-miR-374a-5p	HIF1A	hsa-miR-379-3p	PPARGC1A	hsa-miR-497-5p
ACSL4	hsa-miR-375	HIF1A	hsa-miR-1271-5p	PRDX6	hsa-miR-382-5p
ACSL4	hsa-miR-148b-3p	HIF1A	hsa-miR-1179	PRDX6	hsa-miR-498
ACSL4	hsa-miR-520e	HIF1A	hsa-miR-4735-3p	PRDX6	hsa-miR-671-5p
ACSL4	hsa-miR-519c-5p	HK1	hsa-miR-17-5p	PRDX6	hsa-miR-320b
ACSL4	hsa-miR-524-5p	HK1	hsa-miR-106a-5p	SCD	hsa-miR-16-5p
ACSL4	hsa-miR-545-3p	HK1	hsa-miR-199a-3p	SCD	hsa-miR-215-5p
ACSL4	hsa-miR-576-5p	HK1	hsa-miR-34a-5p	SCD	hsa-miR-218-5p
ACSL4	hsa-miR-582-5p	HK1	hsa-miR-152-3p	SCD	hsa-miR-221-3p
ACSL4	hsa-miR-873-5p	HK1	hsa-miR-185-5p	SCD	hsa-miR-224-5p
ACSL4	hsa-miR-520c-5p	HK1	hsa-miR-34b-5p	SCD	hsa-miR-200b-3p
ACSL4	hsa-miR-320b	HK1	hsa-miR-302d-3p	SCD	hsa-miR-27b-3p
ACSL4	hsa-miR-320c	HK1	hsa-miR-520f-3p	SCD	hsa-miR-185-5p
ACSL4	hsa-miR-224-3p	HK1	hsa-miR-524-5p	SCD	hsa-miR-186-5p
ACSL4	hsa-miR-3144-3p	HK1	hsa-miR-654-5p	SCD	hsa-miR-188-5p
ACSL4	hsa-miR-4295	HSPB1	hsa-miR-1193	SCD	hsa-miR-193a-3p
ACSL4	hsa-miR-4766-5p	LAP3	hsa-miR-26b-5p	SCD	hsa-miR-330-3p
ACSL4	hsa-miR-5688	LAP3	hsa-miR-362-5p	SCD	hsa-miR-324-3p
ACSL4	hsa-miR-1277-5p	LAP3	hsa-miR-656-3p	SCD	hsa-miR-345-5p
ANXA5	hsa-miR-29a-3p	NAMPT	hsa-miR-20a-5p	SCD	hsa-miR-20b-5p
ANXA5	hsa-miR-105-5p	NAMPT	hsa-miR-218-5p	SCD	hsa-miR-495-3p

ANXA5	hsa-miR-193a-3p	NAMPT	hsa-miR-224-5p	SCD	hsa-miR-515-5p
ANXA5	hsa-miR-381-3p	NAMPT	hsa-miR-655-3p	SCD	hsa-miR-510-5p
ANXA5	hsa-miR-1321	NAMPT	hsa-miR-758-3p	SCD	hsa-miR-556-5p
ANXA5	hsa-miR-4735-3p	NAMPT	hsa-miR-340-5p	SCD	hsa-miR-888-5p
BNIP3	hsa-miR-101-3p	NAMPT	hsa-miR-488-3p	SCD	hsa-miR-1287-5p
BNIP3	hsa-miR-27b-3p	NAMPT	hsa-miR-624-3p	SCD	hsa-miR-1270
BNIP3	hsa-miR-1185-5p	NAMPT	hsa-miR-543	SCD	hsa-miR-3126-5p
CTPS1	hsa-miR-135a-5p	NAMPT	hsa-miR-3163	SCD	hsa-miR-4306
CTPS1	hsa-miR-361-3p	NAMPT	hsa-miR-3187-3p	SCD	hsa-miR-4428
EPHA2	hsa-miR-299-3p	NME1	hsa-miR-324-3p	SCD	hsa-miR-4640-3p
EPHA2	hsa-miR-629-5p	NME1	hsa-miR-146b-5p	SCD	hsa-miR-4739
EPHA2	hsa-miR-302e	PCK2	hsa-miR-3150b-3p	SCD	hsa-miR-499b-5p
