# Supplementary material for: Integrated RNA-seq and scRNA-seq to explore the biological mechanisms of mitophagy-related genes in ulcerative colitis
Source: PLoS One. 2026 Apr 20;21(4):e0346974. doi: 10.1371/journal.pone.0346974 (PMC13095012; doi:10.1371/journal.pone.0346974)
Supplement: S6 Table — (PDF) [file pone.0346974.s010.pdf]

Table S6. mRNA-TF interactions.

mRNA	TF	mRNA	TF	mRNA	TF
HK1	CEBPB	NAMPT	SPI1	PGM1	KMT2A
HK1	ESR1	NAMPT	STAT3	PGM1	NRF1
HSPB1	EGR1	NAMPT	TCF7L2	PLOD2	AR
HSPB1	ELF1	NAMPT	AR	PLOD2	CEBPB
HSPB1	ETS1	NAMPT	YY1AP1	PLOD2	EP300
HSPB1	GATA1	NAMPT	CEBPB	PLOD2	ERG
HSPB1	MAX	NAMPT	CREB1	PLOD2	ESR1
HSPB1	MYC	NAMPT	CTCF	PLOD2	FOXA1
HSPB1	NRF1	NME1	JUN	PLOD2	FOXA2
HSPB1	POLR2A	NME1	JUND	PLOD2	HNF4A
HSPB1	TBP	NME1	MAX	PLOD2	HOXB13
HSPB1	ZNF281	NME1	MXI1	PLOD2	NANOG
LAP3	ATF2	NME1	MYC	PLOD2	STAT3
LAP3	CREB1	NME1	MYCN	PPARGC1A	FOXA1
LAP3	CTCF	NME1	NRF1	PPARGC1A	FOXA2
LAP3	E2F6	NME1	POLR2A	PPARGC1A	USF1
LAP3	EGR1	NME1	SMARCA4	PPARGC1A	USF2
LAP3	ELF1	NME1	SMARCC1	PRDX6	BHLHE40
LAP3	ERG	NME1	SPI1	PRDX6	CEBPB
LAP3	ETV1	NME1	STAT3	PRDX6	E2F1
LAP3	GABPA	NME1	TBP	PRDX6	E2F6
LAP3	MAX	NME1	VDR	PRDX6	MAX
LAP3	MXI1	NME1	ZNF24	PRDX6	MNT
LAP3	MYC	NME1	CREBBP	PRDX6	MYC
LAP3	NRF1	NME1	EBF1	PRDX6	NRF1
LAP3	POLR2A	NME1	EGR1	PRDX6	POLR2A
NAMPT	EGR1	NME1	ELF1	PRDX6	SPI1
NAMPT	EP300	NME1	EP300	PRDX6	TBP
NAMPT	FOXA1	NME1	ERG	PRDX6	USF1
NAMPT	FOXA2	NME1	ETS1	PRDX6	USF2
NAMPT	GATA1	NME1	ETV1	SCD	E2F1
NAMPT	GATA2	NME1	FOS	SCD	EBF1
NAMPT	GRHL2	NME1	FOSL1	SCD	MAX
NAMPT	HIF1A	NME1	FOSL2	SCD	MXI1
NAMPT	HOXB13	NME1	FOXA1	SCD	MYC
NAMPT	MAX	NME1	GABPA	SCD	MYCN
NAMPT	BHLHE40	PGM1	CEBPA	SCD	NRF1
NAMPT	MYC	PGM1	CEBPB	SCD	POLR2A
NAMPT	NFKB1	PGM1	EBF1	SCD	TBP
NAMPT	NRF1	PGM1	FOXA2		

TF, Transcription factor.
