# Supplementary material for: Integrated RNA-seq and scRNA-seq to explore the biological mechanisms of mitophagy-related genes in ulcerative colitis
Source: PLoS One. 2026 Apr 20;21(4):e0346974. doi: 10.1371/journal.pone.0346974 (PMC13095012; doi:10.1371/journal.pone.0346974)
Supplement: S7 Table — (PDF) [file pone.0346974.s011.pdf]

Table S7. Univariate and Multivariate logistic regression.

Characteristics	Total (N)	OR (95% CI) Univariate analysis	P value Univariate analysis	OR (95% CI) Multivariate analysis	P value Multivariate analysis
ACAA2	125	36.837 (9.706 - 139.811)	< 0.001	2.425 (0.175 - 33.627)	0.509
BNIP3	125	0.080 (0.027 - 0.236)	< 0.001	0.477 (0.045 - 5.075)	0.539
CKB	125	3.448 (2.038 - 5.834)	< 0.001	5.695 (1.125 - 28.820)	0.035
HIF1A	125	0.029 (0.008 - 0.107)	< 0.001	0.002 (0.000 - 0.880)	0.045
HK1	125	0.012 (0.002 - 0.062)	< 0.001	0.156 (0.004 - 6.246)	0.324
HSPB1	125	12.407 (4.203 - 36.625)	< 0.001	10.153 (0.787 - 131.048)	0.076
MIF	125	0.026 (0.007 - 0.101)	< 0.001	0.396 (0.022 - 7.180)	0.531
NAMPT	125	0.353 (0.209 - 0.597)	< 0.001	19.53 (1.226 - 311.342)	0.035
NME1	125	0.059 (0.020 - 0.177)	< 0.001	0.058 (0.003 - 1.277)	0.071
PCK2	125	8.264 (3.125 - 21.851)	< 0.001	0.009 (0.000 - 0.304)	0.009
PPARGC1A	125	117.641 (17.471 - 792.125)	< 0.001	0.863 (0.005 - 137.139)	0.954
PRDX6	125	37.000 (9.477 - 144.448)	< 0.001	4.766 (0.089 - 254.330)	0.442
SCD	125	0.421 (0.243 - 0.729)	< 0.01	4.394 (1.135 - 17.013)	0.032

OR, odds ratio. CI, confidence interval.
