# Supplementary material for: Integrated RNA-seq and scRNA-seq to explore the biological mechanisms of mitophagy-related genes in ulcerative colitis
Source: PLoS One. 2026 Apr 20;21(4):e0346974. doi: 10.1371/journal.pone.0346974 (PMC13095012; doi:10.1371/journal.pone.0346974)
Supplement: Attachment — Submitted filename: Response to Reviewers. docx.docx [file pone.0346974.s014.docx]

Response Letter

Your comments, along with those of the other reviewers, are extremely important to our article. We greatly appreciate your insights, which have significantly contributed to enhancing the quality of our work. In this revision, we have prepared a detailed point-by-point response letter for you and the other valued reviewers. This letter acknowledges your feedback and clearly outlines the revisions we have made.

| **Date:** | Sep 28 2025 11:40PM |
| --- | --- |
| **To:** | "Yan Qi" qiyankm@163.com |
| **From:** | "PLOS ONE" plosone@plos.org |
| **Subject:** | PLOS ONE Decision: Revision required [PONE-D-25-44869] |

PONE-D-25-44869

Integrated RNA-seq and scRNA-seq to explore the biological mechanisms of mitophagy-related genes in ulcerative colitis

PLOS ONE

Dear Dr. Qi,

Thank you for submitting your manuscript to PLOS ONE. After careful consideration, we feel that it has merit but does not fully meet PLOS ONE’s publication criteria as it currently stands. Therefore, we invite you to submit a revised version of the manuscript that addresses the points raised during the review process.

Please submit your revised manuscript by Nov 12 2025 11:59PM. If you will need more time than this to complete your revisions, please reply to this message or contact the journal office at[plosone@plos.org](mailto:plosone@plos.org). When you're ready to submit your revision, log on to https://www.editorialmanager.com/pone/ and select the 'Submissions Needing Revision' folder to locate your manuscript file.

Please include the following items when submitting your revised manuscript:

- A rebuttal letter that responds to each point raised by the academic editor and reviewer(s). You should upload this letter as a separate file labeled 'Response to Reviewers'.
- A marked-up copy of your manuscript that highlights changes made to the original version. You should upload this as a separate file labeled 'Revised Manuscript with Track Changes'.
- An unmarked version of your revised paper without tracked changes. You should upload this as a separate file labeled 'Manuscript'.

If you would like to make changes to your financial disclosure, please include your updated statement in your cover letter. Guidelines for resubmitting your figure files are available below the reviewer comments at the end of this letter.

If applicable, we recommend that you deposit your laboratory protocols in protocols.io to enhance the reproducibility of your results. Protocols.io assigns your protocol its own identifier (DOI) so that it can be cited independently in the future. For instructions see:[https://journals.plos.org/plosone/s/submission-guidelines#loc-laboratory-protocols](https://journals.plos.org/plosone/s/submission-guidelines#loc-laboratory-protocols). Additionally, PLOS ONE offers an option for publishing peer-reviewed Lab Protocol articles, which describe protocols hosted on protocols.io. Read more information on sharing protocols at<https://plos.org/protocols?utm_medium=editorial-email&utm_source=authorletters&utm_campaign=protocols>.

We look forward to receiving your revised manuscript.

Kind regards,

Alexis G. Murillo Carrasco
Academic Editor
PLOS ONE

Journal Requirements:

When submitting your revision, we need you to address these additional requirements.

1. Please ensure that your manuscript meets PLOS ONE's style requirements, including those for file naming. The PLOS ONE style templates can be found at

https://journals.plos.org/plosone/s/file?id=wjVg/PLOSOne_formatting_sample_main_body.pdf and

<https://journals.plos.org/plosone/s/file?id=ba62/PLOSOne_formatting_sample_title_authors_affiliations.pdf>

**Comment 1: Thank you for your careful review and valuable comments.We will rigorously verify all submitted documents against PLOS ONE's formatting guidelines and templates, ensuring proper naming conventions and content formatting to guarantee full compliance with the journal's latest requirements. Thank you again for your valuable suggestion.**

2. Thank you for stating the following financial disclosure:

“The National Natural Science Foundation of China (Grant number [81960868]) and the Yunnan Provincial Science and Technology Department, funded by the Applied Basic Research Joint SpecialFunds of Yunnan University of Chinese Medicine (Grant numbers [202001AZ070001-051] and [202101AZ070001-013]), High-level TCM talents: (reserve talents) project of Yunnan (2021. No. 1), Xingdian Talent Support Program-Youth Talent Special Project (2023. No. 166), Yunnan Provincial Science and Technology Department - Yunnan University of Traditional Chinese Medicine Joint special Project (Grant numbers [202201AG070192]), Applied Basic Research Key Project of Yunnan (202501AS070155), Yunnan Applied Basic Research Project--Joint Project of Traditional Chinese Medicine and Surface(202001AZ070001-051)”.

Please state what role the funders took in the study. If the funders had no role, please state: 'The funders had no role in study design, data collection and analysis, decision to publish, or preparation of the manuscript.'

If this statement is not correct you must amend it as needed.

Please include this amended Role of Funder statement in your cover letter; we will change the online submission form on your behalf.

**Comment Financial Disclosure:**

**Thank you for your careful review and valuable comments. I have determined that all of the items mentioned in the financial disclosure provide financial support for this article. The funders had no role in study design, data collection and analysis, decision to publish, or preparation of the manuscript. Thank you again for your valuable suggestion.**

3. When completing the data availability statement of the submission form, you indicated that you will make your data available on acceptance. We strongly recommend all authors decide on a data sharing plan before acceptance, as the process can be lengthy and hold up publication timelines. Please note that, though access restrictions are acceptable now, your entire data will need to be made freely accessible if your manuscript is accepted for publication. This policy applies to all data except where public deposition would breach compliance with the protocol approved by your research ethics board. If you are unable to adhere to our open data policy, please kindly revise your statement to explain your reasoning and we will seek the editor's input on an exemption. Please be assured that, once you have provided your new statement, the assessment of your exemption will not hold up the peer review process.

**Comment: Thank you for your careful review of my manuscript.I confirm that the data will be made available after the paper is accepted.**

4. PLOS ONE now requires that authors provide the original uncropped and unadjusted images underlying all blot or gel results reported in a submission’s figures or Supporting Information files. This policy and the journal’s other requirements for blot/gel reporting and figure preparation are described in detail at https://journals.plos.org/plosone/s/figures#loc-blot-and-gel-reporting-requirements and https://journals.plos.org/plosone/s/figures#loc-preparing-figures-from-image-files. When you submit your revised manuscript, please ensure that your figures adhere fully to these guidelines and provide the original underlying images for all blot or gel data reported in your submission. See the following link for instructions on providing the original image data: [https://journals.plos.org/plosone/s/figures#loc-original-images-for-blots-and-gels.](https://journals.plos.org/plosone/s/figures#loc-original-images-for-blots-and-gels.)

In your cover letter, please note whether your blot/gel image data are in Supporting Information or posted at a public data repository, provide the repository URL if relevant, and provide specific details as to which raw blot/gel images, if any, are not available. Email us at plosone@plos.org if you have any questions.

**Comment 4: Thank you for your careful review of my manuscript. I have read the relevant requirements of this journal for protein immunoassay, and I have uploaded the original image of protein immunoassay as a Supporting Information according to the submission requirements, In the cover letter, blot image data has been clearly written as supporting information to be uploaded.**

5. If the reviewer comments include a recommendation to cite specific previously published works, please review and evaluate these publications to determine whether they are relevant and should be cited. There is no requirement to cite these works unless the editor has indicated otherwise.
   **Comment 5: Thank you for your careful review of my manuscript. I have carefully reviewed this manuscript, and the reviewer did not raise any questions about the references. Thank you again for your valuable suggestions.**

Additional Editor Comments:

Please revise all reviewers' comments and respond to them accordingly. In addition, please be sure to describe if you applied p-adjustment for multiple comparisons (for example, for generating the volcano plot). If so, please include the strategy applied (BH, FDR, Others).

According to the figure shown in the Nomogram, there is no clear reference to the risk related to these markers. Perhaps it is relevant to test the MRG-based score in combination with other clinical profiles instead of combining all relevant genes? How can it be applied to the routine? In the current Figure 6D, it is hard to see the contribution of each gene, particularly in the cases of PPARGC1A and MIF. Please revise it.

Please describe in high-level details the methods followed to annotate potential cell types in the single-cell dataset. How did you define which cell types could be present in your sample? Following the previous comment, I suggest showing a dotplot with relevant genes from Figure 9, organized by cell type and donor type (UC or control).

Finally, please add statistical comparisons in Figure 10C and attach the melting curves for SYBR-amplified regions.

[Note: HTML markup is below. Please do not edit.]

Reviewers' comments:

Reviewer's Responses to Questions

**Comments to the Author**

1. Is the manuscript technically sound, and do the data support the conclusions?

The manuscript must describe a technically sound piece of scientific research with data that supports the conclusions. Experiments must have been conducted rigorously, with appropriate controls, replication, and sample sizes. The conclusions must be drawn appropriately based on the data presented.

Reviewer #1: Yes

Reviewer #2: Yes

Reviewer #3: Yes

2. Has the statistical analysis been performed appropriately and rigorously?

Reviewer #1: Yes

Reviewer #2: Yes

Reviewer #3: No

3. Have the authors made all data underlying the findings in their manuscript fully available?

The[PLOS Data policy](http://www.plosone.org/static/policies.action#sharing)requires authors to make all data underlying the findings described in their manuscript fully available without restriction, with rare exception (please refer to the Data Availability Statement in the manuscript PDF file). The data should be provided as part of the manuscript or its supporting information, or deposited to a public repository. For example, in addition to summary statistics, the data points behind means, medians and variance measures should be available. If there are restrictions on publicly sharing data—e.g. participant privacy or use of data from a third party—those must be specified.

Reviewer #1: Yes

Reviewer #2: Yes

Reviewer #3: Yes

4. Is the manuscript presented in an intelligible fashion and written in standard English?

PLOS ONE does not copyedit accepted manuscripts, so the language in submitted articles must be clear, correct, and unambiguous. Any typographical or grammatical errors should be corrected at revision, so please note any specific errors here.

Reviewer #1: Yes

Reviewer #2: Yes

Reviewer #3: Yes

5. Review Comments to the Author

Please use the space provided to explain your answers to the questions above. You may also include additional comments for the author, including concerns about dual publication, research ethics, or publication ethics. (Please upload your review as an attachment if it exceeds 20,000 characters)

Reviewer #1: In this manuscript, Ma et al. identified 13 key genes involved in ulcerative colitis through bioinformatic analysis of publicly available next-generation DNA sequencing raw data. Furthermore, the authors validated their observations on a bench using a mouse model of ulcerative colitis. The strengths of the study design are as follows: 1) Gene prioritization was performed using RNA-seq data from human tissues, narrowing down to hub and key genes; 2) single-cell RNA-seq data were used; and 3) expression changes in mice were confirmed by RT-qPCR and Western blotting. However, mechanistic verification is lacking in research design. In other words, experiments such as using the identified gene knock-out mice, knock-down experiment using siRNA, or experiments using inhibitors of the identified protein. However, the reviewer has never requested additional experiments for any review. Such so-called "Reviewer Experiments" are, in fact, not very meaningful in academic papers (Nature 2011;472:391). The authors should construct their logic and draw conclusions based solely on the data presented. Furthermore, most PLOS ONE values represent the validity and robustness of the methodology. Therefore, reviewers should point out the following aspects concerning the validity of the methods rather than the significance of the author's research results.

Overall, the proposed methodology is reasonable. However, as the authors themselves indicate in the title, the most controversial point is the integration of RNA-seq and scRNA-seq results as well as subsequent gene prioritization. There is still no established consensus on this methodology, with various reports and debates. The authors should discuss this issue in the Discussion section.

**Comment: Thank you for your careful review and valuable comments. Regarding the controversial issues you raised, there is currently no unified standard in this field, and related analytical methods are continuously evolving and improving. In this study, we first systematically screened differentially expressed genes, co-expression modules, and key genes at the bulk RNA-seq level, then combined single-cell transcriptomic data for cell-type-specific localization and functional annotation. This strategy, adopted in multiple high-level studies, can reflect cellular heterogeneity while maintaining overall tissue expression characteristics. We will supplement the analysis of methodological controversies in the discussion section of the revised manuscript, citing recent research advancements to further illustrate the rationale and future directions of this workflow. Specific modifications are detailed in the Discussion section on page 27, line 617-633. Thank you again for your valuable suggestion.**

Reviewer #2: Ma et al aims to decipher the mechanism of mitophagy related genes in ulcerative colitis. Overall, it is a well designed, executed and validated work. Multi-modal tools were utilized to expand spectrum of evidence representation.

Here are some points to note:

1. It is understandable that the diagnostic model developed by the authors is still in the early stage, given that more datasets have to be used for cross-validation. Could the authors deliberate more of the potential use, as well as the sensitivity and specificity of limitation of detection?
**Comment: Thank you for your careful review and valuable comments. The multi-gene diagnostic model developed in this study demonstrated high accuracy in distinguishing ulcerative colitis (UC) from healthy controls, with an AUC of 0.974, indicating strong diagnostic potential. This model is expected to serve as a molecular tool for early clinical diagnosis, identification of complex cases, and personalized risk assessment, thereby enhancing the precision of UC diagnosis. Current evaluations of model sensitivity and specificity primarily rely on internal validation within public databases. However, detection outcomes are influenced by factors such as sample sources and sequencing platforms, necessitating additional independent cohort data and multi-center studies to further validate their generalization capabilities and clinical diagnostic efficacy. We plan to expand sample types and testing methods in subsequent research, while refining model performance through multi-center, prospective studies to facilitate its clinical translation. Thank you again for your valuable suggestion.**

2. Following point 1, what kind of human samples to be needed for such a test? Turnaround duration from sample to data, and to diagnostic result? Estimated cost per test? Benefit population figures from such a test of UC patients?

**Comment: Thank you for your careful review and valuable comments. The detection model proposed in this study is primarily based on colon tissue samples obtained through standard clinical colonoscopy biopsies, following the same histopathological examination protocol used for ulcerative colitis. In both the manuscript and research analysis, we employed colon tissue samples for transcriptome sequencing and qRT-PCR to detect key gene expression levels. The relevant data and analytical procedures are thoroughly described in the Materials and Methods and Results sections of the manuscript. At present, based on the existing experimental process, the fastest time to complete the sample collection, RNA extraction, detection and diagnostic model results is 2-4 days, and it is expected to be further shortened to 24 hours if more simple detection methods such as quantitative PCR or chip are used. The cost of a single test is preliminarily estimated at 500-1000 RMB based on different testing methods. With large-scale clinical implementation, the testing cost is expected to decrease further. This detection method is applicable to all suspected or confirmed ulcerative colitis patients, demonstrating potential value in various clinical scenarios including disease classification, activity assessment, and treatment monitoring. Benefiting a broad population, it shows promising clinical translation prospects. In future work, we will optimize the testing process and evaluate its clinical performance by incorporating more actual samples and prospective evaluations. Thank you again for your valuable suggestion.**

Reviewer #3: Dear Authors,

I think that this manuscript could benefit from a substantial revision - because of the points below, the main flow of the argument is made difficult to follow.

Main points:
1. References in the text to figures and tables are sometimes incorrect and confused, making the flow of the text difficult to follow. In particular:

- Line 248 - Supplementary Figure S1 is not a flowchart of the study, but bar plots of gene ontology analyses

**Comment: Thank you for your careful review and valuable comments. We will carefully review and standardize the references and descriptions of charts in the full text based on your suggestions, ensuring their content aligns with the main text to enhance the article's logic and readability. Specific modifications are detailed on page 11, line 253. Thank you again for your valuable suggestion.**

- Line 257 states 21665 DEGs, but there are only 1802 shown in figure 2B (this might be a misreading on my part) - and also the reference to the comparison between MRGs and DEGs should refer to figure 2B rather than 2A

**Comment: Thank you for your careful review and valuable comments. The figure 21,665 in the text refers to the total number of genes detected in the UC dataset, while 1,802 represents the significantly differentially expressed genes screened through differential analysis that meet the threshold criteria (|logFC|> 0.5 and P <0.05). These two metrics have distinct statistical definitions and will be explicitly distinguished throughout the paper. Regarding figure numbering: the volcano plot (Figure 2A) illustrates the overall distribution of all differentially expressed genes, while the Venn diagram (Figure 2B) shows the overlap between these genes and master regulatory genes (MRGs). We will revise the figure numbering in the revised manuscript to ensure clarity and accuracy. For detailed modifications, please refer to page 12, line 267-270. Thank you again for your valuable suggestion.**

- Line 311-317 - the genes in figure S3A are ALDH6A1, ACADSB, ALDH2, PC, ALDH1A1, and ACSL1 but the text around these lines does not refer to these, but to other datasets

**Comment: Thank you for your careful review and valuable comments. Regarding your inquiry, we have reviewed the manuscript and analysis workflow, confirming that the mRNA-miRNA interaction network in Supplementary Fig. S3A is indeed based on genes such as ALDH6A1, ACADSB, ALDH2, PC, ALDH1A1, and ACSL1, rather than the 20 hub genes mentioned in the main text. In the subsequent revision, we will reorganize and redraw Supplementary Fig. S3A to align with the hub genes referenced in the main text, while synchronously revising the relevant text and supplementary tables to ensure complete correspondence between figures, text, and descriptions. Specific modifications are detailed on page 14, lines 317-328. Thank you again for your valuable suggestion.**

- Lines 325-335 refer to cluster analysis for the genes, but this is not labelled on the plot in 4B

**Comment: Thank you for your careful review and valuable comments.Regarding your feedback, Figure 4B in this study presents the expression correlation heatmap of 20 hub genes in the UC group. The correlation analysis results are detailed in lines 325-335 of the main text. Based on the clustering results from the heatmap, these 20 genes were grouped into three clusters. The biological significance of these groupings is further illustrated by the chord diagram (Fig. 4A) and functional similarity analysis (Fig. 4C). Figure 4B primarily visualizes inter-gene correlations, clearly demonstrating the analytical approach and key findings. We appreciate your suggestions. To further enhance the intuitiveness of result presentation, we will consider adding more intuitive annotations to cluster groups in the correlation heatmaps in future work, enabling readers to quickly grasp clustering information.**

**Thank you again for your valuable suggestion.**

- Line 343/figure 4G - the figure contains genes not written in the text here.

**Comment: Thank you for your careful review and valuable comments. To address the issue of the genes in Figure 4G not being included in the main text, we will review and verify the relevant content during revisions to ensure the chart presentation aligns with the core gene list, thereby avoiding potential confusion. Specific modifications are detailed at page 15, lines 348 and 355. Thank you again for your valuable suggestion.**

- Line 317 - Table S5 in the text is actually Table S6
- Line 323 - Table S6 in the text is actually Table S7
- Line 391 - Table S7 in the text is actually Table S8

**Comment: Thank you for your careful review and valuable comments. We have carefully verified the correspondence between the Supplementary Table numbers and the main text, and further ensured their consistency in the revised draft for reader convenience. Thank you for your careful review and valuable comments.**

- Line 408-409 mentioned 15000 variable genes in the scRNA analysis, but the figure 7B shows 2000 variable genes

**Comment: Thank you for your careful review and valuable comments. Regarding the 15,000 and 2,000 highly variable genes, we selected 15,000 for PCA and subsequent dimensionality reduction clustering analysis during the actual analysis process, as described in the Materials and Methods section of the manuscript (see page 9, lines 188-189 for specific modifications). Figure 7B displays the top 2000 highly variable genes, the default visualization threshold set by the Seurat package for clear and intuitive presentation. This approach does not compromise the core analysis findings. To ensure clarity, we will provide additional explanations in the revised manuscript. Specific modifications are detailed on page 18, lines 426-428. Thank you again for your valuable suggestion.**

- Lines 429-430 have the labels for figures 8B and 8C swapped in the text

**Comment: Thank you for your careful review and valuable comments. Regarding the sequence of Figures 8B and 8C in the main text description, we will verify and correct the relevant content in the revised draft to ensure consistency between the legend and the main text description, avoiding any misalignment between the figure captions and the content. For specific modifications, please refer to page 19, lines 441-443. Thank you again for your valuable suggestion.**
- Also Figure 8D is referred to as a heat map, but it is a plot of the expression of seven chosen genes shown on the UMAP
**Comment: Thank you for your careful review and valuable comments. In the manuscript, we presented the expression patterns of seven signature genes in the single-cell UMAP visualization space, which is more accurately termed a 'UMAP signature expression map' rather than a 'heat map'. We will revise this description in the revised draft to accurately reflect the content of Figure 8D. The specific modifications are detailed on page 19, lines 437-438 and 446. Thank you again for your valuable suggestion.**

2. Supplementary Figure S1 and Supplementary Table S5 both report gene ontology analysis results but are not referred to in the text, in the methods, results or the discussion. If this analysis was performed on this data and is to be presented in the manuscript, then it should be reported on and discussed in the context of MRGs and/or ulcerative colitis.
**Comment: Thank you for your careful review and valuable comments. GO and KEGG analyses were incorporated into our preliminary bioinformatics workflow as standard procedures. However, given the study's primary focus on key gene networks, immune infiltration, and their diagnostic significance, the corresponding analyses were not elaborated or thoroughly examined in the main body's Materials and Methods, Results, or Discussion sections. To maintain content focus and avoid redundancy, we have removed GO and KEGG-related tables and figures from the supplementary materials. Specific modifications are detailed in the supplementary materials section. Thank you again for your valuable suggestion.**

3. Figure 6D shows the nomogram for the predictor - I'm not certain what the point of this diagram is in the manuscript. What if the ranges of expression of these genes are outside the values on the scales? Do we take the minimum or the maximum of the point score? How does the linear predictor and the risk get calculated from the values here?
**Comment: Thank you for your careful review and valuable comments. The nomogram, serving as a visualization tool for the multi-factor logistic regression model in this study, provides an individualized risk prediction method based on the expression levels of 13 key genes. Specific modifications are detailed on page 8, lines 181-182. For cases where gene expression values exceed the nomogram's scale range, it is recommended in practice to use the maximum or minimum values of the scale axis to ensure the model's continuity and stability. This should be clearly marked in the figure legend of Figure 6D in the main text. By mapping gene expression levels to corresponding scores and summing them, the nomogram calculates risk probabilities on the total score axis, thereby establishing a correspondence with the linear predictors of the logistic regression model. Specific modifications are detailed on page 18, lines 416-418. Thank you again for your valuable suggestion.**

4. The main figures are low resolution (as they currently stand, maybe a high resolution version is available?). This particularly affects figure 9A and 9B where the gene labels are difficult to read because of resolution and the compression artefacts.
**Comment: Thank you for your careful review and valuable comments. In the revised draft, we will further optimize the resolution and label layout of key images, particularly Figures 9A and 9B, and provide high-definition original images in the text to enhance clarity and readability. Specific modifications are detailed in page 33-44, lines773-784-xx.Thank you again for your valuable suggestion.**

5. The materials and methods appear to consistently refer to raw p-values. It it clear from some of the later text in the results that some of these have been adjusted for multiple comparisons - this is not mentioned in the methods section and should be made clear here.
**Comment: Thank you for your careful review and valuable comments. In this study, preliminary screening in differential expression analysis and correlation analysis was primarily based on raw P-values. For functional enrichment analyses such as GSEA, the Benjamini-Hochberg method was employed to correct for multiple comparisons, with the corrected q-values (FDR) serving as the significance criterion. We will provide further clarification and supplementation in the revised manuscript regarding the specific applications of P-values and multiple corrections (q-values/FDR) in all statistical analyses, ensuring clear and accurate descriptions to facilitate readers' understanding of the analytical procedures and result evaluation standards. Specific revisions are detailed on page 11, lines 239-241. Thank you again for your valuable suggestion.**

Some more specific points to make:
1. Figure 1C - PC2 is shown as explaining 'a5.8%' of the variance.

**Comment: Thank you for your careful review and valuable comments. Regarding the labeling of the PC2 variance explanation ratio in Figure 1C, we will revise 'a5.8%' to '5.8%' in the subsequent draft and conduct a thorough check and proofreading of the coordinate axis labels on the relevant PCA plots to ensure accurate representation. For specific modifications, please refer to page 33, line 762. Thank you again for your valuable suggestion.**

2. In figure 2C, what is the score that is plotted in the heatmap?

**Comment: Thank you for your careful review and valuable comments. The 'score' in Figure 2C represents the standardized gene expression levels of each mitochondrial autophagy-related differentially expressed gene (MRDEGs) across samples. The group comparisons are based on ulcerative colitis (UC) and control groups, providing an intuitive visualization of gene expression patterns and statistical differences. To avoid ambiguity, we will add further clarifications to the legend and methods section in the revised manuscript. Specific modifications are detailed on page 12, lines 277-279. Thank you again for your valuable suggestion.**

3. Figure 2D has the word 'adataset' (possible typo) in the title. There is also no label on the red-white-blue scale

**Comment: Thank you for your careful review and valuable comments. Regarding your inquiry, we will verify and correct the issue in the revised draft. Additionally, to address the absence of the red-white-blue gradient scale on the right side of the heatmap, we will explicitly indicate in the legend of Figure 2D that red, white, and blue represent high, medium, and low expression levels, respectively. For detailed modifications, please refer to page 12, lines 280-281. Thank you again for your valuable suggestion.**

4. Figure 5B/line 358-359: place the labels on figure 5B in the order they are in the text, or write the text in the same order as the labels on Figure 5B

**Comment: Thank you for your careful review and valuable comments. We have aligned the description sequence of immune cells in the main text with the label order in Figure 5B, and standardized the terminology to ensure clearer correspondence between text and figures. Specific modifications are detailed on page 16, lines 363-365. We appreciate your valuable suggestions.**

5. Figure 6C - this would be better shown as log-HRs on the x axis because of the scales on the x axis. This would make the difference between the calculated CIs and the HR 0 line clearer to see

**Comment: Thank you for your careful review and valuable comments. Regarding the presentation of Figure 6C, we currently use HR as the x-axis, primarily because this approach allows clinical readers to intuitively understand the impact intensity of individual genes, while also aligning with common visualization practices in the field. We recognize that using log(HR) as the x-axis can more clearly demonstrate the relationship between confidence intervals and the HR=0 line. In future analyses with expanded sample sizes or more in-depth studies, we will actively consider adopting the log(HR) scale to further enhance the visual clarity and academic rigor of the charts. Thank you again for your valuable suggestion.**

6. Figure 10A - label on the colon images which is the diseased and which is the normal.

**Comment: Thank you for your careful review and valuable comments. To address the suggestion regarding the grouping labels in Figure 10A, we will explicitly add the labels "Normal (NC)" and "ulcerative colitis (UC)" in the revised draft's captions. The images and captions will further clarify the locations of lesions and normal tissues to enhance visual clarity and readability. Specific modifications are detailed on pages 21 and 42, lines 490-492 and 782. Thank you again for your valuable suggestion.**

7. Figure 11 - order the datastet by placing the significant genes first: also place the gene name as a title on each pair of bars for the bar plot

**Comment: Thank you for your careful review and valuable comments. For the visualization proposal in Figure 11, we will rank the 13 genes in the revised draft according to statistical significance, with gene names as captions above each column pair to better highlight key expression differences and significance. Detailed modifications are provided on page 43, line 783. Thank you again for your valuable suggestion.**

8. Figure 12A - What are the two bands that appear on the blot for the MIF protein? Which one has been taken as the one to quantify?

**Comment: Thank you for your careful review and valuable comments. Regarding the two MIF protein bands in Fig. 12A, based on the protein molecular weight and relevant literature, the lower molecular weight band (approximately 12-13 kDa) represents the monomeric form of MIF, which is the primary focus of expression and analysis in this study. The upper band may correspond to a dimer or post-translational modification product. In the quantitative protein analysis, we exclusively analyzed the lower monomeric band to ensure result accuracy and comparability. Specific modifications are detailed in page 22, 44, line 510-511, 784. Thank you again for your valuable suggestion.**

9. Figure 12B - this would benefit from the individual blot quantifications in the two conditions shown on each bar - the blot images show significant variation (eg NAMPT in the NC condition, and the top MIF band in the UC condition). It is also not mentioned what the intensity was quantified relative to.

**Comment: Thank you for your careful review and valuable comments. In this Western blot quantitative analysis, β-actin was used as an internal reference for normalization, effectively reflecting protein expression trends and inter-group differences. Regarding the visualization of individual sample data points, considering the current research focus and experimental conditions, we believe the existing presentation method adequately meets data expression and analysis requirements. Future studies will further diversify data visualization approaches to enhance interpretability. Thank you again for your valuable suggestion.**

6. PLOS authors have the option to publish the peer review history of their article ([what does this mean?](https://journals.plos.org/plosone/s/editorial-and-peer-review-process#loc-peer-review-history)). If published, this will include your full peer review and any attached files.

If you choose “no”, your identity will remain anonymous but your review may still be made public.

**Do you want your identity to be public for this peer review?**For information about this choice, including consent withdrawal, please see our[Privacy Policy](https://www.plos.org/privacy-policy).

Reviewer #1:**Yes:**Shigekazu Sugino

Reviewer #2: No

Reviewer #3: No

[NOTE: If reviewer comments were submitted as an attachment file, they will be attached to this email and accessible via the submission site. Please log into your account, locate the manuscript record, and check for the action link "View Attachments". If this link does not appear, there are no attachment files.]

While revising your submission, please upload your figure files to the Preflight Analysis and Conversion Engine (PACE) digital diagnostic tool, <https://pacev2.apexcovantage.com/>. PACE helps ensure that figures meet PLOS requirements. To use PACE, you must first register as a user. Registration is free. Then, login and navigate to the UPLOAD tab, where you will find detailed instructions on how to use the tool. If you encounter any issues or have any questions when using PACE, please email PLOS at[figures@plos.org](mailto:figures@plos.org). Please note that Supporting Information files do not need this step.

**Comment: Thank you for your careful inspection and patient guidance. I have registered PACE, and used it to complete the technical identification and inspection of the pictures, and uploaded the correct format of PACE export to the submission system of this journal to ensure that the pictures meet the submission requirements.**

In compliance with data protection regulations, you may request that we remove your personal registration details at any time.[(Remove my information/details)](https://www.editorialmanager.com/pone/login.asp?a=r). Please contact the publication office if you have any questions.
