# Supplementary material for: Integrated RNA-seq and scRNA-seq to explore the biological mechanisms of mitophagy-related genes in ulcerative colitis
Source: PLoS One. 2026 Apr 20;21(4):e0346974. doi: 10.1371/journal.pone.0346974 (PMC13095012; doi:10.1371/journal.pone.0346974)
Supplement: Attachment — Submitted filename: Response to Reviewers-2.docx [file pone.0346974.s015.docx]

Response Letter

We would like to express our sincere gratitude to you and the reviewers for your valuable comments on our manuscript. In light of the thorough and insightful feedback provided, we have revised the manuscript accordingly. We have added relevant content to address the reviewers' suggestions, and we have rewritten several sections to ensure clarity and completeness. We hope that these modifications meet the reviewers' expectations and enhance the overall quality of the manuscript.

PONE-D-25-44869R2

Integrated RNA-seq and scRNA-seq to explore the biological mechanisms of mitophagy-related genes in ulcerative colitis

PLOS One

Dr Yan Qi

We've checked your submission and before we can proceed, we need you to address the following issues:

1. Please note that PLOS ONE requires submissions reporting blot or gel data to comply in full with the reporting requirements described at https://journals.plos.org/plosone/s/figures#loc-blot-and-gel-reporting-requirements. We now require authors to provide the original unadjusted and uncropped images for any blot or gel data reported in PLOS ONE submissions. In our internal checks for your submission, we noted that you did not provide original raw image files supporting blot/gel data in response to our previous request.

all blot and gel figures at this time; see the above URL for instructions on how the raw blot/gel image data should be prepared and submitted. Note that we cannot consider your manuscript further until you have met this requirement. If there are any blot/gel figures in your manuscript or its Supporting Information for which you cannot provide the original uncropped and unadjusted images please specify this in your response and provide details as to which figures are affected by raw image data unavailability. Email us at plosone@plos.org if you have questions.

**Comment: Thank you for your thorough review and valuable feedback. The original raw image files containing blot/gel data are available in Supplementary Material Fig S4. and is noted in Fig. 12 of the manuscript body. The full membrane data has been provided as requested. Please refer to it.**

2. If possible, please upload a file showing your changes either highlighted or using track changes. This should be uploaded as a Revised Manuscript w/tracked changes, file type. Please follow this link for more information: http://blogs.PLOS.org/everyone/2011/05/10/how-to-submit-your-revised-manuscript/

We've returned your manuscript to your account. Please resolve these issues and resubmit your manuscript within 21 days. If you need more time, please email the journal office at plosone@plos.org. We are happy to grant extensions of up to one month past this due date. If we do not hear from you within 21 days, we will withdraw your manuscript.

**Comment: Thank you for your reminder. The manuscript with revision marks has been re-uploaded, and the modified parts are highlighted in red. Please upload the file as a revised version.**

Please log on to PLOS Editorial Manager at https://www.editorialmanager.com/pone/ to access your manuscript. You will find your manuscript in the 'Submissions Sent Back to Author' link under the New Submissions menu. Be sure to remove your previous manuscript file if you are uploading a new file in response to these requests. After you've made the changes requested above, please be sure to view and approve the revised PDF after rebuilding the PDF to complete the resubmission process.

We are requesting these changes to comply with the PLOS ONE submission guidelines (https://journals.plos.org/plosone/s/submission-guidelines). Please note that we won't send your manuscript for review until you have resolved the above requests.

Thank you for submitting your work to PLOS ONE and supporting our mission of Open Science.

Kind regards,

Trisha Mae Tañedo Perez

PLOS One

__________________________________________________

In compliance with data protection regulations, you may request that we remove your personal registration details at any time. (Use the following URL: https://www.editorialmanager.com/pone/login.asp?a=r). Please contact the publication office if you have any questions.
